# PlantNLRatlas: a comprehensive dataset of full- and partial-length NLR resistance genes across 100 chromosome-level plant genomes

**DOI:** 10.3389/fpls.2023.1178069

**Published:** 2023-04-14

**Authors:** Xiang Li, Linna Ma, Yingmin Wang, Chen Ye, Cunwu Guo, Yingbin Li, Xinyue Mei, Fei Du, Huichuan Huang

**Affiliations:** ^1^ State Key Laboratory for Conservation and Utilization of Bio-Resources in Yunnan, Yunnan Agricultural University, Kunming, China; ^2^ Key Laboratory for Agro-Biodiversity and Pest Control of Ministry of Education, Yunnan Agricultural University, Kunming, China

**Keywords:** genome-wide analysis, NB-LRR, NLRs, R gene, PlantNLRatlas, bioinformatics

## Abstract

Plants have evolved two layers of protection against biotic stress: PAMP-triggered immunity (PTI) and effector-triggered immunity (ETI). The primary mechanism of ETI involves nucleotide-binding leucine-rich repeat immune receptors (NLRs). Although NLR genes have been studied in several plant species, a comprehensive database of NLRs across a diverse array of species is still lacking. Here, we present a thorough analysis of NLR genes across 100 high-quality plant genomes (PlantNLRatlas). The PlantNLRatlas includes a total of 68,452 NLRs, of which 3,689 are full-length and 64,763 are partial-length NLRs. The majority of NLR groups were phyletically clustered. In addition, the domain sequences were found to be highly conserved within each NLR group. Our PlantNLRatlas dataset is complementary to RefPlantNLR, a collection of NLR genes which have been experimentally confirmed. The PlantNLRatlas should prove helpful for comparative investigations of NLRs across a range of plant groups, including understudied taxa. Finally, the PlantNLRatlas resource is intended to help the field move past a monolithic understanding of NLR structure and function.

## 1 Introduction

Plants have evolved two layers of defense against biotic and abiotic stressors. Pathogen-associated molecular patterns (PAMPs) or damage-associated molecular patterns (DAMPs) trigger the first layer of defense, PAMP-triggered immunity (PTI), *via* cell surface localized pattern recognition receptors (PRRs) (Jones and Dangl, 2006). As a countermeasure, numerous pathogens, including bacteria, fungi, oomycetes, and nematodes, transfer virulence-associated molecules into plant cells or the apoplast. These molecules, such as effectors released by the bacterial type III secretion system (T3SS), act to curtail host immune response and promote pathogenic invasion and proliferation (Xin et al., 2018; Rocafort et al., 2020). Direct or indirect detection of effectors by plant nucleotide-binding leucine-rich repeat immune receptors (NLRs) activates the second, more robust layer, of defense: effector-triggered immunity (ETI) (Jones and Dangl, 2006). NLRs can be divided into three subclasses according to their characteristic N-terminal domain: toll/interleukin-1 receptor-like (TIR), coiled coil (CC), or resistance to powdery mildew 8 (RPW8). Accordingly, the three classes have been named TIR-NBS-LRR (TNL), CC-NBS-LRR (CNL), and RPW8-NBS-LRR (RNL), respectively (Mchale et al., 2006). Recent research suggests that partial-length NLRs may be crucial for plant defense (Wu et al., 2022), although relatively few studies have focused on these partial-length genes (Andolfo et al., 2022).

The quality and availability of reference datasets are of great importance to computational biology studies. To date, only a few NLR datasets have been published and made available for analysis. The largest collection of experimentally verified NLR proteins from 73 plants, which includes 415 NLRs, is the RefPlantNLR database (Kourelis et al., 2021). The NLRscape webserver contains a curated collection of over 80,000 proteins containing NOD-like receptor signatures, as identified in UniProtKB (Martin et al., 2022). Because of improvements in sequencing techniques and bioinformatics methods, and with the advent of third generation sequencing, many more accurately-assembled plant genomes are being published (Sharma et al., 2022). Utilizing these high-quality plant genomes alongside other sequencing methods, researchers are discovering ever more NLRs (Inturrisi et al., 2020; Lee et al., 2021; Huang et al., 2022). However, most of these studies involve only one plant species, even though a sea of plant genomes is available. To bolster efforts to develop stress-resistant crops, it is imperative to identify and validate NLRs across an array of plant species at a large scale.

Here, we sought to identify full- and partial-length NLRs by analyzing 100 high-quality plant genomes. In all, we identified 68,452 distinct NLR genes across 15 subgroups, including four full-length and eleven partial-length NLR groups. Phylogenetic analysis indicated that the matching domain sequence is highly conserved for each NLR group, with each group containing numerous subclades. Comparison of our dataset with RefPlantNLR suggested that there are still many NLRs requiring experimental validation. Based on the results of this study, we created the PlantNLRatlas dataset (**Supplementary Table 2**), which includes information about the 100 plant species surveyed, identified domains, and relevant literature. Our PlantNLRatlas dataset offers a comprehensive complement to RefPlantNLR to aid in the identification and cloning of NLRs.

## 2 Materials and methods

### 2.1 Genomic data processing

We downloaded the genomic sequences and annotation files (gff format) for 100 plant species, utilizing the most recent version of each genome (**Supplementary Table 1**). Protein FASTA sequences were generated using gffread (v0.11.7) (Pertea and Pertea, 2020). Each plant was classified as either a eudicot or monocot according to the NCBI Taxonomy database (Schoch et al., 2020). The RefPlantNLR protein sequences were downloaded from the Zenodo database (https://zenodo.org/record/3936022#.Y3OolHbMLiA) (Kourelis et al., 2021).

### 2.2 Domain annotation

OrthoFinder (v2.5.4) (Emms and Kelly, 2019) was used, with default parameters, to create the phylogenetic tree utilizing the NLRs domain sequences in order to investigate the phylogenetical relationships between the 100 plants.

Protein sequences were annotated with Pfam identifiers using InterProScan (v5.56-89.0) (Jones et al., 2014), with parameters -f TSV -app Pfam. The IPS2fpGs.sh script (https://github.com/AndolfoG/HRP) (Andolfo et al., 2022) was used to classify the NB-LRR genes as either full- or partial-length according to the domain(s) they contained. We used the homology-based R-gene prediction pipeline to identify new NB-LRR genes (Andolfo et al., 2022) and to annotate the RefPlantNLR protein sequences. NLR-Annotator (v2) (Steuernagel et al., 2020), with default parameters, was used to identify NLR genes using CDS sequences that were either downloaded or parsed using gffread (v0.11.7).

### 2.3 Phylogenetic analysis

Based on the InterProScan results, domain sequences were parsed from the corresponding protein sequence of each identified NB-LRR gene. If a gene contained more than one domain, we spliced the domains in the order in which they appeared in the gene sequence. Domain sequences were aligned using Clustal Omega (v 1.2.4) (Sievers et al., 2014), with default parameters. After alignment, we used FastTree (v2.1.10) (Price et al., 2010) to construct the phylogenetic tree with parameter -lg. The R packages ggtree (v3.6.0) (Yu et al., 2017) and ggtreeExtra (v1.8.0) (Xu et al., 2021) were used to visualize the phylogenetic tree.

### 2.4 Protein sequence analysis

The protein length, molecular weight, and isoelectric point were calculated using ExPASy (https://www.expasy.org/) (Artimo et al., 2012). The results were visualized using the R package ggplot2 (v3.4.0) (Wickham, 2016).

### 2.5 RNA-Seq data processing

To study the expression patterns of NLR genes, we utilized transcriptomic data from chitin- or flg22-treated polyploid wheat (*Triticum aestivum*; accession number PRJEB23056) (Ramírez-González et al., 2018) and chitin-treated soybean (*Glycine max*; accession number PRJNA594515) (Yao et al., 2020). HISAT2 (v2.2.1) (Kim et al., 2019) was used to create a genomic index and align the raw fastq data to the genome, using default parameters. Gene counts were calculated from the sorted bam file using featureCounts (v2.0.2) (Liao et al., 2013). The expression patterns of selected NLR genes were visualized using the pheatmap package (v1.0.12) in R.

## 3 Results

### 3.1 Identification of NLR genes and creation of the PlantNLRatlas dataset

**Figure 1:**
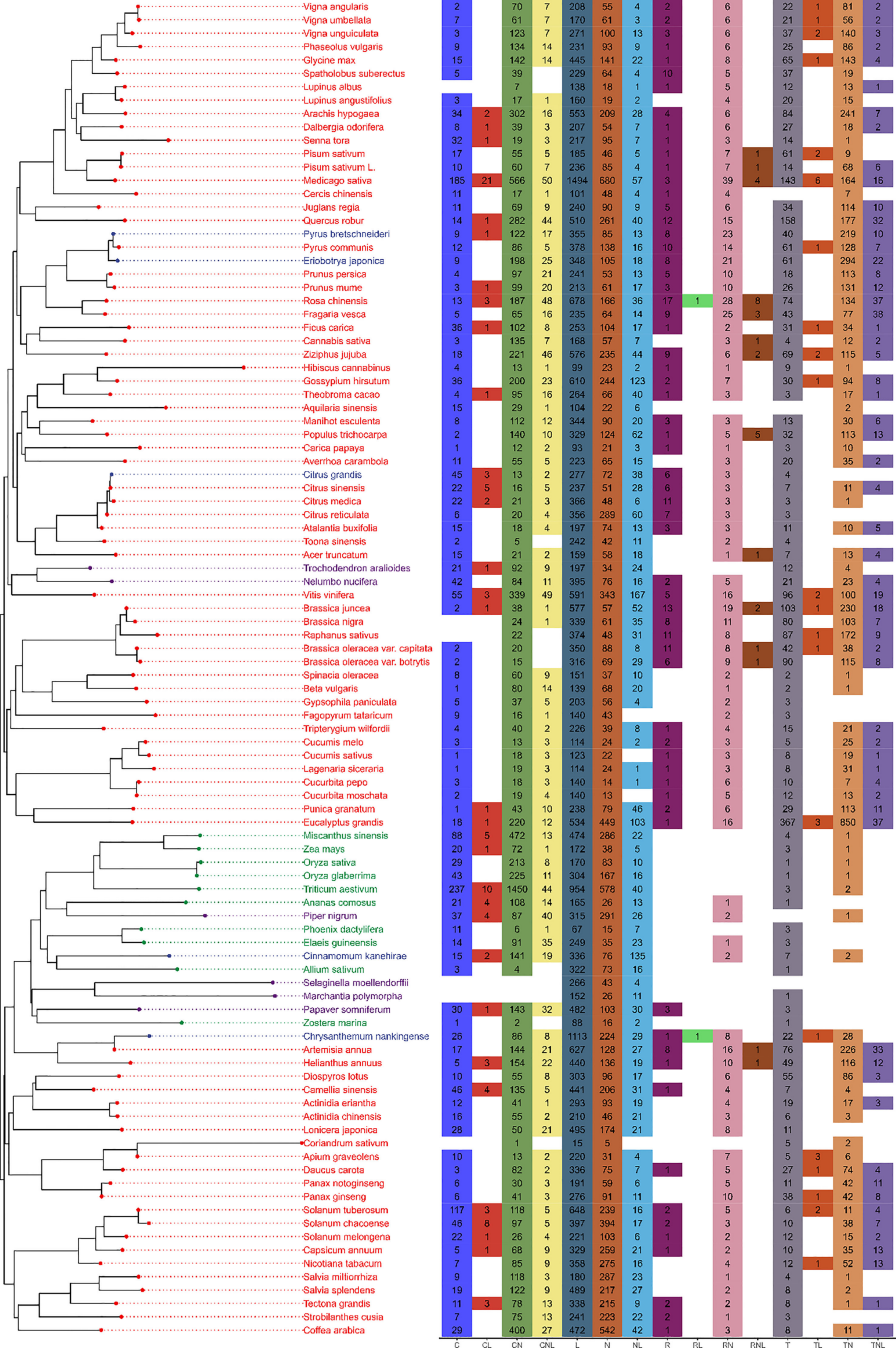
Survey of NLRs across 100 plant genomes. The left panel shows the phylogenetic tree created utilizing NLR domain sequences. Eudicots, monocots, and other plants are represented by green, red, and blue letters, respectively. The right panel shows the NRL gene groups. Numbers in each cell indicate the number of NLRs identified in each plant genome. Blank cells indicate that no NLRs were discovered in the plant genome.

A total of 68,452 full- or partial-length NBS-LRR resistance genes were identified across 100 plant genomes, including 83 eudicots, 10 monocots, and 7 other plants representing 31 orders and 48 families (**Figure 1**, **Supplementary Table 2**). On average, there were 685 NLRs per genome. Coriander (*Coriandrum sativum*) (Song et al., 2020), a medicinal herb and spice plant, contained the least NLR genes (28). Alfalfa (*Medicago sativa*) (Chen et al., 2020), an important forage crop, contained the most NLR genes (3,428). Two groups of domains, L (Leucine Rich repeat) and N (NB-ARC domain), were identified across all 100 genomes. The L group was the most abundant, with 31,481 family members. The RL (Arabidopsis broad-spectrum mildew resistance protein RPW8 and Leucine Rich repeat) group contained only two members, present in *Rosa chinensis* (Raymond et al., 2018) and *Chrysanthemum nankingense* (Song et al., 2018). One of the 30-gene full-length groups, RNL was present in 14 species belonging to 6 orders and 8 families. Half of these 14 species are important food crops, including pea (*Pisum sativum*) (Yang et al., 2022), mustard (*Brassica juncea*) (Yang et al., 2016), cauliflower (*Brassica oleracea* var. botrytis) (Belser et al., 2018), cabbage (*Brassica oleracea* var. capitata) (Cai et al., 2020), alpine strawberry (*Fragaria vesca*) (Shulaev et al., 2011), sunflower (*Helianthus annuus*) (Badouin et al., 2017), and jujube (*Ziziphus jujuba*) (Liu et al., 2014). The largest full-length group, CNL, was present in 89 species and contained 1,010 genes. The last group of NLRs contained 474 genes and was present in 59 species of non-monocots, which is consistent with previous research (Shao et al., 2016; Andolfo et al., 2019). In contrast to the norm of classifying each gene into one NLR group, we found that 520 genes contained multiple NLR domains (**Supplementary Table 3**). These genes were present in 45 species, including wheat (*T. aestivum*; 61 genes), corn (*Zea mays*; 1 gene), and African rice (*Oryza glaberrima*; 18 genes).

**Figure 2:**
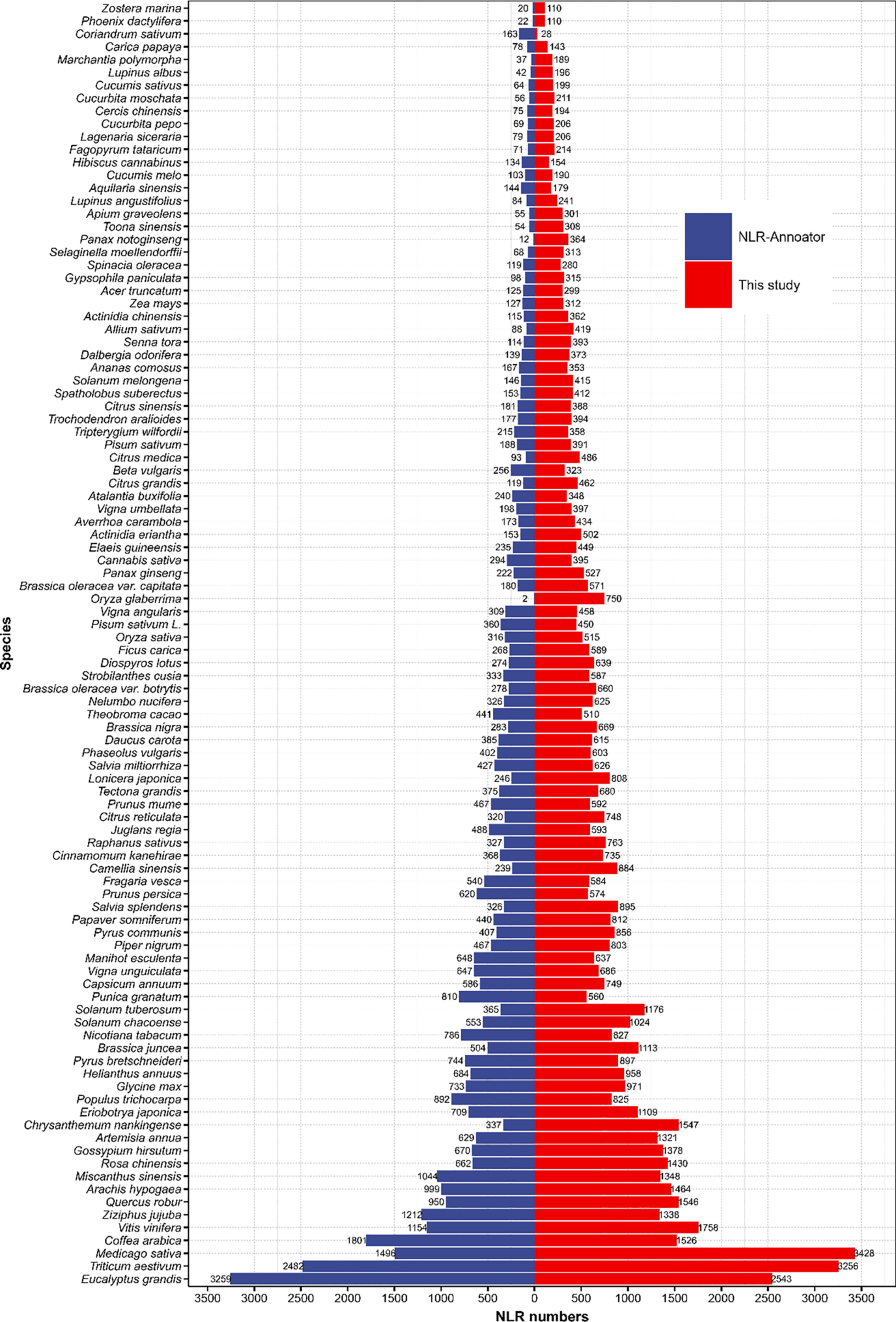
Comparison of NLR genes discovered using both our pipeline and NLR-Annotator. The blue bar shows the number of NLRs identified by NLR-Annotator. The red bar shows the number of NLRs identified by our pipeline.

In order to validate our results, we used NLR-Annotator to identify NLRs from across 100 plant genomes. With the exception of seven plants (*Eucalyptus grandis*, *Coffea arabica*, *Punica granatum*, *Coriandrum sativum*, *Populus trichocarpa*, *Prunus persica*, and *Manihot esculenta*), our pipeline led to the discovery of more NLRs than were identified by NLR-Annotator (**Figure 2**). For example, in the commercially important hardwood species *Eucalyptus grandis*, our pipeline identified 2,543 NLRs while NLR-Annotator identified 3,259 NLRs. A previous study on *Eucalyptus grandis* utilized the Hidden Markov Model approach to identify 1,487 NLRs (Christie et al., 2016). Additionally, NLR-Annotator identified two NLRs in *Oryza glaberrima*, while our pipeline identified 750 NLRs.

Our dataset of 68,452 NBS-LRR resistance genes (**Figure 1**) was used to create the PlantNLRatlas (**Supplementary Table 2**). The dataset was expanded to include pertinent details about each genome, including species, domain information, and relevant web links. Each species identifier included the scientific name, Chinese name, Order, Family, Genus, and Species. Domain information included protein ID, corresponding gene ID, NLR group, domain starting and ending positions (via InterProScan), database name and ID (via InterProScan), protein sequence, and extracted domain sequences. Relevant literature was annotated within the “Paper.link” column, and downloadable genomes were annotated within the “Download.link” column. Finally, the primary process code was made available on GitHub (https://github.com/lixiang117423/PlantNLRatlas).

### 3.2 PlantNLRatlas is complementary to RefPlantNLR

**Figure 3:**
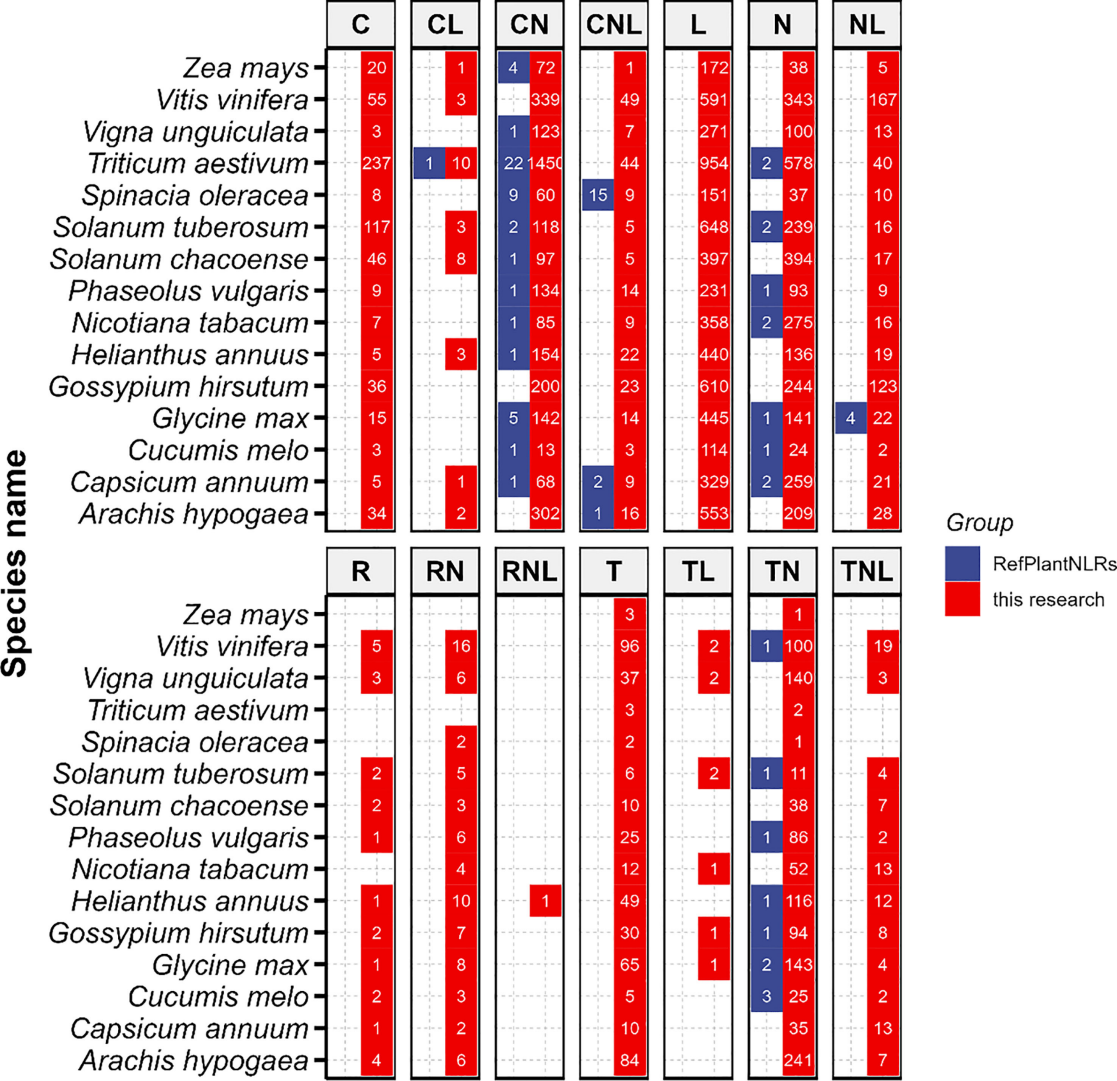
Comparison of NLR genes identified in this study with those in RefPlantNLR. Genes in RefPlantNLR are represented in blue, and genes in our study are represented in red. Numbers in each cell indicate the number of NLRs identified in each plant genome. Blank cells indicate that no NLRs were discovered in the plant genome.

We compared our results to RefPlantNLR, a comprehensive collection of experimentally validated plant disease resistance proteins from the NLR family (Kourelis et al., 2021). First, we classified each protein into a corresponding NLR group by annotating the protein sequences from RefPlantNLR using our pipeline. Next, we compared the number of NLR genes in 15 species using both the RefPlantNLR dataset and our pipeline (**Figure 3**). According to the RefPlantNLR dataset, the 15 species were classified into six NLR groups (CL, CN, CNL, N, NL, and TN). Compared to our dataset, the RefPlantNLR dataset contained fewer NLR genes among the six groups, suggesting that there are still many NLR genes which have not yet been identified and cloned. For wheat (*Triticum aestivum*), RefPlantNLR contained 22 experimentally validated NLRs, while our dataset included 1,450 NLRs. In the oilseed sunflower (*Helianthus annuus*), only one NLR gene was identified. The majority of experimentally validated NLRs within RefPlantNLR contained more than one NLR domain, with the exception of group N. For full-length NLRs within RefPlantNLR, group NL was found to contain four NLRs and group CNL was found to contain 18 NLRs.

**Figure 4:**
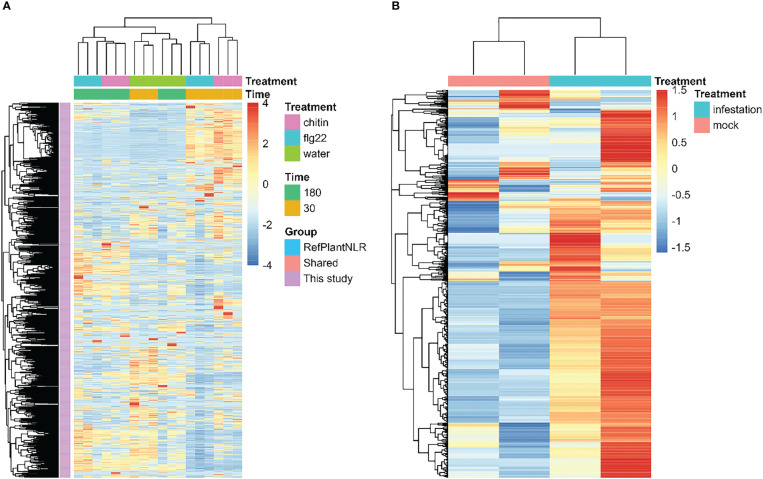
**(A)**: Wheat (*Triticum aestivum*) NLR gene expression patterns according to both RefPlantNLR and PlantNLRatlas. The heatmap was created using FPKM (fragments per kilobase of transcript per million mapped reads) values. Genes identified in both RefPlantNLR and PlantNLRatlas are annotated as “shared NLR genes”. **(B)**: Heatmap of soybean (*Glycine max*) NLR gene expression patterns in response to challenge by soybean aphid (*Aphis glycines*), according to PlantNLRatlas.

To study the expression patterns of NLR genes in response to PTI and ETI, we utilized RNA-Seq data from polyploid wheat (*Triticum aestivum*) treated with either chitin or flg22 (Ramírez-González et al., 2018). Overall, similar expression patterns were observed for NLR genes from both PlantNLRatlas and RefPlantNLR, particularly in the first cluster containing four cloned NLRs (**Figure 4A**). It is likely that many of the NLR genes identified in the PlantNLRatlas dataset may share similar functions in the wheat immune response, including in PTI and ETI. These results suggest that many NLR genes await experimental validation and cloning. Furthermore, the soybean NLR genes reported in our dataset were all significantly upregulated when plants were challenged by the soybean aphid (*Aphis glycines*) (**Figure 4B**), suggesting that these genes may play important roles in the plant response to herbivory. Overall, our PlantNLRatlas dataset is likely to be an invaluable asset for the continued study of plant NLRs.

### 3.3 NLR gene characteristics across 100 plants

We evaluated the characteristics of each NLR gene, including gene length (protein sequence length), protein molecular weight, and protein isoelectric point (**Supplementary Figures 1B–D**). As expected, full-length NLR genes were the longest and had the greatest molecular weight (**Supplementary Figures 1B, D**). Group T NLRs were the shortest and had the lowest molecular weight. Group TNL NLRs were the longest and had the greatest molecular weight. The average protein length and molecular weight were 722 amino acids and 81.5 kDa, respectively. The smallest NLR protein was from black mustard (*Brassica nigra*; 34 amino acids and 3.7 kDa) while the largest was from kenaf (*Hibiscus cannabinus*; 5,849 amino acids and 680.7 kDa). The average NLR protein isoelectric point was 6.8 in dicots and 7.1 in monocots, and ranged from 3.2 in rose (*Rosa chinensis*) to 12.9 in black pepper (*Piper nigrum*). The isoelectric points of 12 NLR groups were < 7, indicating that the majority of NLR proteins are acidic. Additionally, the average isoelectric points of the four full-length NLR groups were < 7 (**Supplementary Figure 1D**).

### 3.4 Multiple sequence alignment and phylogenetic analysis

**Figure 5:**
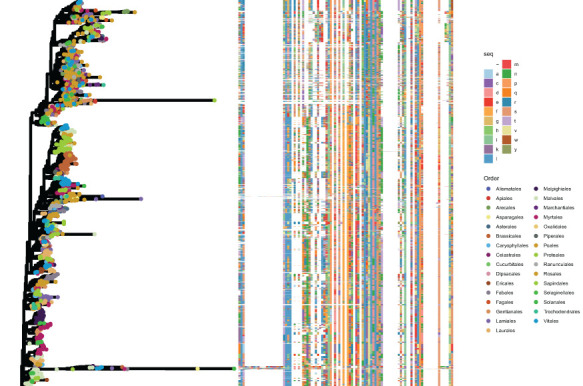
Phylogenetic analysis of group NL. The left panel shows the phylogenetic tree created using FastTree, with different colors denoting different orders. The right panel shows the corresponding aligned domain sequences, with different colors corresponding to different amino acids.

**Figure 6:**
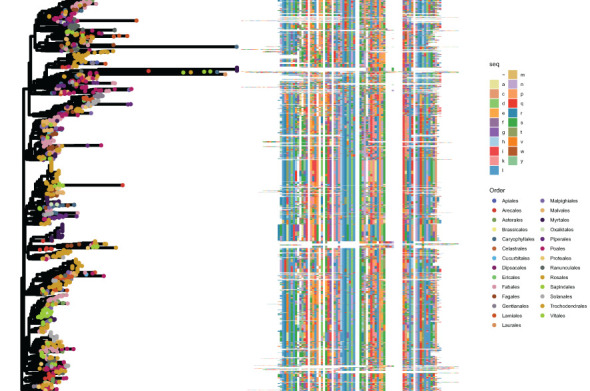
Phylogenetic analysis of group CNL. The left panel shows the phylogenetic tree created using FastTree, with different colors denoting different orders. The right panel shows the corresponding aligned domain sequences, with different colors corresponding to different amino acids.

**Figure 7:**
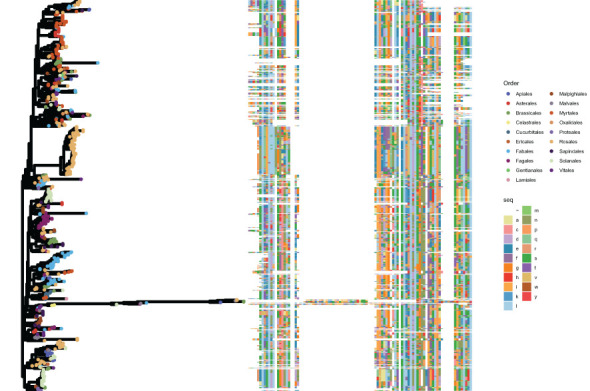
Phylogenetic analysis of group TNL. The left panel shows the phylogenetic tree created using FastTree, with different colors denoting different orders. The right panel shows the corresponding aligned domain sequences, with different colors corresponding to different amino acids.

The domain sequences were extracted from each NLR protein sequence *via* InterProScan, and then aligned to construct a phylogenetic tree (**Figures 5**–**7**, **Supplementary Figures 2** **–12**). Because some NLR genes can be classified into several NLR groups according to the domains present therein, the domain sequences of all transcripts of each gene were used to construct the phylogenetic tree. Overall, the domain sequences within each NLR group were highly conserved (**Figures 3**–**5**, **Supplementary Figures 2** **–12**). Several NLR groups were divided into multiple subclades, particularly group T (**Supplementary Figure 2**), group C (**Supplementary Figure 4**), and group N (**Supplementary Figure 6**). Furthermore, several NLR groups were phyletically clustered according to order, including group T (**Supplementary Figure 2**) and group TN (**Supplementary Figure 11**). However, the four full-length NLR genes did not clearly cluster into order-based subclades.

## 4 Discussion

As the world population continues to grow, the global food supply is becoming more vulnerable. Pathogens and pests, in particular, present significant threats to crop production. In response to these challenges, plants have developed powerful defense mechanisms to combat diseases throughout their entire life cycle (Jones and Dangl, 2006). The NLR genes represent one aspect of plant defense. With the publication of many high-quality plant genomes and advancements in sequencing technology and bioinformatics, new NLR genes are being continuously discovered and cloned (Kourelis et al., 2021). Until recently, the majority of NLR genes were believed to fall into three main subgroups: TIR-NBS-LRR (TNL), CC-NBS-LRR (CNL), and RPW8-NBS-LRR(RNL) (Ding et al., 2020; Zhang et al., 2022). However, a recent study found that partial-length NLRs may be essential to plant defense (Wu et al., 2022). Here, we surveyed NLR genes across 100 high-quality plant genomes, including from 83 eudicots, 10 monocots, and 7 other plants belonging to 31 orders and 48 families. The 68,452 identified NLR genes were split into 15 subgroups, 4 of which contained full-length NLRs and 11 of which contained partial-length NLRs.

Modern biological research is often conducted with large datasets and databases, such as those provided by NCBI or EMBEL (Madeira et al., 2022). For studies of NLRs, RefPlantNLR and NLRscape are the two most commonly utilized databases (Kourelis et al., 2021; Martin et al., 2022). RefPlantNLR contains 481 experimentally verified NLRs and NLRscape contains over 80,000 protein sequences identified *via* UniProtKB. Here, we have created a novel and complementary reference dataset, PlantNLRatlas. Although the RefPlantNLR contains several highly significant plant species, we have expanded our dataset to include an even wider variety of useful plants, including several traditional Chinese medicinal (TCM) plants such as ginseng (*Panax notoginseng*) (Chen et al., 2017), and major crops such as wheat, rice, and corn/maize. Our 100PlantsRNLs dataset contains significantly more NLR genes than RefPlantNLR, indicating that many NLR genes are in need of experimental validation and cloning.

Many bioinformatic analyses utilize only one representative transcript per gene (Yang et al., 2021; Zhao et al., 2021). Here, we discovered that 520 NLR genes from 45 plants could be classified as either partial- or full-length NLRs (**Supplementary Table 3**), including genes from *Oryza glaberrima* (18 NLRs), *Triticum aestivum* (61 NLRs), and *Zea mays* (1 NLR). These results suggest that, as a best practice, bioinformatic analyses and experimental validations should take into account the structural and functional variations of each transcript derived from a single gene.

While many studies have probed the evolutionary history of NLR genes as a group, few have specifically focused on how individual NLR genes have evolved across various plants (Kourelis et al., 2021; Martin et al., 2022). Here, we performed phylogenetic analyses for each distinct NLR group. Because each NLR group was found to contain one or more subclades, we can infer that each group has a unique evolutionary history. Additionally, the majority of NLRs clustered into groups according to the order to which each species belonged, further indicating that each group has a unique evolutionary history.

## 5 Conclusion

In conclusion, this study is the first to conduct a genome-wide scan of plant NLR genes using the high-quality genomes of 100 plant species. In all, we identified 3,689 full-length NLRs genes and 64,763 partial-length NLRs genes. Based on this, we created a dataset with a wealth of information that may be used to complement RefPlantNLR, the most comprehensive dataset available at the time. Furthermore, we used transcriptomic data to confirm the identified NLR genes, and we discovered that several of the NLR genes may be crucial for the plant response to biotic stress. Our study and the corresponding dataset will be a valuable tool for NLR gene research and will support the discovery of plant stress resistance genes.

## Data availability statement

The original contributions presented in the study are included in the article/**Supplementary Material**. Further inquiries can be directed to the corresponding author.

## Author contributions

XL and HH raised scientific questions and designed the study. XL, LM, and YW collected data. XL and HH performed the analysis. XL, YW, LM, CY, CG, YL, XM, and FD wrote the manuscript. All authors read and approved the final manuscript.

## Supplementary material

Supplementary Table 1 Metadata of the 100 plants used in this study.

Supplementary Table 2 The PlantNLRatlas dataset.

Supplementary Table 3 The subset of one gene divided into multiple NLR gene groups.

Supplementary Figure 1 Number of genes within and characteristics of each NLR group. Different colors represent full-length and partial-length genes. **(A)**: Number of genes in each group. **(B)**: Protein lengths. **(C)**: Protein molecular weights, with the red point indicating the average value. **(D)**: Protein isoelectric points, with the red point indicating the average value.

Supplementary Figure 2 Phylogenetic analysis of group T. The left panel shows the phylogenetic tree created using FastTree, with different colors denoting different orders. The right panel shows the corresponding aligned domain sequences, with different colors corresponding to different amino acids.

Supplementary Figure 3 Phylogenetic analysis of group R. The left panel shows the phylogenetic tree created using FastTree, with different colors denoting different orders. The right panel shows the corresponding aligned domain sequences, with different colors corresponding to different amino acids.

Supplementary Figure 4 Phylogenetic analysis of group C. The left panel shows the phylogenetic tree created using FastTree, with different colors denoting different orders. The right panel shows the corresponding aligned domain sequences, with different colors corresponding to different amino acids.

Supplementary Figure 5 Phylogenetic analysis of group L. The left panel shows the phylogenetic tree created using FastTree, with different colors denoting different orders. The right panel shows the corresponding aligned domain sequences, with different colors corresponding to different amino acids.

Supplementary Figure 6 Phylogenetic analysis of group N. The left panel shows the phylogenetic tree created using FastTree, with different colors denoting different orders. The right panel shows the corresponding aligned domain sequences, with different colors corresponding to different amino acids.

Supplementary Figure 7 Phylogenetic analysis of group RN. The left panel shows the phylogenetic tree created using FastTree, with different colors denoting different orders. The right panel shows the corresponding aligned domain sequences, with different colors corresponding to different amino acids.

Supplementary Figure 8 Phylogenetic analysis of group CL. The left panel shows the phylogenetic tree created using FastTree, with different colors denoting different orders. The right panel shows the corresponding aligned domain sequences, with different colors corresponding to different amino acids.

Supplementary Figure 9 Phylogenetic analysis of group CN. The left panel shows the phylogenetic tree created using FastTree, with different colors denoting different orders. The right panel shows the corresponding aligned domain sequences, with different colors corresponding to different amino acids.

Supplementary Figure 10 Phylogenetic analysis of group TL. The left panel shows the phylogenetic tree created using FastTree, with different colors denoting different orders. The right panel shows the corresponding aligned domain sequences, with different colors corresponding to different amino acids.

Supplementary Figure 11 Phylogenetic analysis of group TN. The left panel shows the phylogenetic tree created using FastTree, with different colors denoting different orders. The right panel shows the corresponding aligned domain sequences, with different colors corresponding to different amino acids.

Supplementary Figure 12 Phylogenetic analysis of group RNL. The left panel shows the phylogenetic tree created using FastTree, with different colors denoting different orders. The right panel shows the corresponding aligned domain sequences, with different colors corresponding to different amino acids.

## References

[B1] Andolfo G. Di Donato A. Chiaiese P. De Natale A. Pollio A. Jones J. D. G.. (2019). Alien domains shaped the modular structure of plant NLR proteins. Genome Biol. Evol. 11, 3466–3477. doi: 10.1093/gbe/evz248 31730154 PMC7145615

[B2] Andolfo G. Dohm J. C. Himmelbauer H. (2022). Prediction of NB-LRR resistance genes based on full-length sequence homology. Plant J. 110, 1592–1602. doi: 10.1111/tpj.15756 35365907 PMC9322396

[B3] Artimo P. Jonnalagedda M. Arnold K. Baratin D. Csardi G. De Castro E.. (2012). ExPASy: SIB bioinformatics resource portal. Nucleic Acids Res. 40, W597–W603. doi: 10.1093/nar/gks400 22661580 PMC3394269

[B4] Badouin H. Gouzy J. Grassa C. J. Murat F. Staton S. E. Cottret L.. (2017). The sunflower genome provides insights into oil metabolism, flowering and asterid evolution. Nature 546, 148–152. doi: 10.1038/nature22380 28538728

[B5] Belser C. Istace B. Denis E. Dubarry M. Baurens F.-C. Falentin C.. (2018). Chromosome-scale assemblies of plant genomes using nanopore long reads and optical maps. Nat. Plants 4, 879–887. doi: 10.1038/s41477-018-0289-4 30390080

[B6] Cai X. Wu J. Liang J. Lin R. Zhang K. Cheng F.. (2020). Improved brassica oleracea JZS assembly reveals significant changing of LTR-RT dynamics in different morphotypes. Theor. Appl. Genet. 133, 3187–3199. doi: 10.1007/s00122-020-03664-3 32772134

[B7] Chen W. Kui L. Zhang G. Zhu S. Zhang J. Wang X.. (2017). Whole-genome sequencing and analysis of the Chinese herbal plant panax notoginseng. Mol. Plant 10, 899–902. doi: 10.1016/j.molp.2017.02.010 28315474

[B8] Chen H. Zeng Y. Yang Y. Huang L. Tang B. Zhang H.. (2020). Allele-aware chromosome-level genome assembly and efficient transgene-free genome editing for the autotetraploid cultivated alfalfa. Nat. Commun. 11, 2494. doi: 10.1038/s41467-020-16338-x 32427850 PMC7237683

[B9] Christie N. Tobias P. A. Naidoo S. Külheim C. (2016). The eucalyptus grandis NBS-LRR gene family: physical clustering and expression hotspots. Front. Plant Sci. 6. doi: 10.3389/fpls.2015.01238 PMC4709456 26793216

[B10] Ding L. Xu X. Kong W. Xia X. Zhang S. Liu L.-W.. (2020). Genome-wide identification and expression analysis of rice NLR genes responsive to the infections of xanthomonas oryzae pv. oryzae and magnaporthe oryzae. Physiol. Mol. Plant Pathol. 111, 101488. doi: 10.1016/j.pmpp.2020.101488

[B11] Emms D. M. Kelly S. (2019). OrthoFinder: phylogenetic orthology inference for comparative genomics. Genome Biol. 20, 238. doi: 10.1186/s13059-019-1832-y 31727128 PMC6857279

[B12] Huang Z. Qiao F. Yang B. Liu J. Liu Y. Wulff B. B. H.. (2022). Genome-wide identification of the NLR gene family in haynaldia villosa by SMRT-RenSeq. BMC Genomics 23, 118. doi: 10.1186/s12864-022-08334-w 35144544 PMC8832786

[B13] Inturrisi F. Bayer P. E. Yang H. Tirnaz S. Edwards D. Batley J. (2020). Genome-wide identification and comparative analysis of resistance genes in brassica juncea. Mol. Breed. 40, 78. doi: 10.1007/s11032-020-01159-z

[B14] Jones P. Binns D. Chang H.-Y. Fraser M. Li W. Mcanulla C.. (2014). InterProScan 5: genome-scale protein function classification. Bioinformatics 30, 1236–1240. doi: 10.1093/bioinformatics/btu031 24451626 PMC3998142

[B15] Jones J. D. G. Dangl J. L. (2006). The plant immune system. Nature 444, 323–329. doi: 10.1038/nature05286 17108957

[B16] Kim D. Paggi J. M. Park C. Bennett C. Salzberg S. L. (2019). Graph-based genome alignment and genotyping with HISAT2 and HISAT-genotype. Nat. Biotechnol. 37, 907–915. doi: 10.1038/s41587-019-0201-4 31375807 PMC7605509

[B17] Kourelis J. Sakai T. Adachi H. Kamoun S. (2021). RefPlantNLR is a comprehensive collection of experimentally validated plant disease resistance proteins from the NLR family. PloS Biol. 19, e3001124. doi: 10.1371/journal.pbio.3001124 34669691 PMC8559963

[B18] Lee H.-Y. Mang H. Choi E. Seo Y.-E. Kim M.-S. Oh S.. (2021). Genome-wide functional analysis of hot pepper immune receptors reveals an autonomous NLR clade in seed plants. New Phytol. 229, 532–547. doi: 10.1111/nph.16878 32810286 PMC7756659

[B19] Liao Y. Smyth G. K. Shi W. (2013). featureCounts: an efficient general purpose program for assigning sequence reads to genomic features. Bioinformatics 30, 923–930. doi: 10.1093/bioinformatics/btt656 24227677

[B20] Liu M.-J. Zhao J. Cai Q.-L. Liu G.-C. Wang J.-R. Zhao Z.-H.. (2014). The complex jujube genome provides insights into fruit tree biology. Nat. Commun. 5, 5315. doi: 10.1038/ncomms6315 25350882 PMC4220462

[B21] Madeira F. Pearce M. Tivey A. R. N. Basutkar P. Lee J. Edbali O.. (2022). Search and sequence analysis tools services from EMBL-EBI in 2022. Nucleic Acids Res. 50, W276–W279. doi: 10.1093/nar/gkac240 35412617 PMC9252731

[B22] Martin E. C. Ion C. F. Ifrimescu F. Spiridon L. Bakker J. Goverse A.. (2022). NLRscape: an atlas of plant NLR proteins. Nucleic Acids Res. 51 (D1), D1470–D1482. doi: 10.1093/nar/gkac1014 PMC9825502 36350627

[B23] Mchale L. Tan X. Koehl P. Michelmore R. W. (2006). Plant NBS-LRR proteins: adaptable guards. Genome Biol. 7, 212. doi: 10.1186/gb-2006-7-4-212 16677430 PMC1557992

[B24] Pertea G. Pertea M. (2020). GFF utilities: GffRead and GffCompare [version 2; peer review: 3 approved]. F1000Research 9, 304. doi: 10.12688/f1000research.23297.1 PMC7222033 32489650

[B25] Price M. N. Dehal P. S. Arkin A. P. (2010). FastTree 2 – approximately maximum-likelihood trees for Large alignments. PloS One 5, e9490. doi: 10.1371/journal.pone.0009490 20224823 PMC2835736

[B26] Ramírez-González R. H. Borrill P. Lang D. Harrington S. A. Brinton J. Venturini L.. (2018). The transcriptional landscape of polyploid wheat. Science 361, eaar6089. doi: 10.1126/science.aar6089 30115782

[B27] Raymond O. Gouzy J. Just J. Badouin H. Verdenaud M. Lemainque A.. (2018). The Rosa genome provides new insights into the domestication of modern roses. Nat. Genet. 50, 772–777. doi: 10.1038/s41588-018-0110-3 29713014 PMC5984618

[B28] Rocafort M. Fudal I. Mesarich C. H. (2020). Apoplastic effector proteins of plant-associated fungi and oomycetes. Curr. Opin. Plant Biol. 56, 9–19. doi: 10.1016/j.pbi.2020.02.004 32247857

[B29] Schoch C. L. Ciufo S. Domrachev M. Hotton C. L. Kannan S. Khovanskaya R.. (2020). NCBI taxonomy: a comprehensive update on curation, resources and tools. Database 2020, baaa062. doi: 10.1093/database/baaa062 32761142 PMC7408187

[B30] Shao Z.-Q. Xue J.-Y. Wu P. Zhang Y.-M. Wu Y. Hang Y.-Y.. (2016). Large-Scale analyses of angiosperm nucleotide-binding site-Leucine-Rich repeat genes reveal three anciently diverged classes with distinct evolutionary patterns. Plant Physiol. 170, 2095–2109. doi: 10.1104/pp.15.01487 26839128 PMC4825152

[B31] Sharma P. Masouleh A. K. Topp B. Furtado A. Henry R. J. (2022). De novo chromosome level assembly of a plant genome from long read sequence data. Plant J. 109, 727–736. doi: 10.1111/tpj.15583 34784084 PMC9300133

[B32] Shulaev V. Sargent D. J. Crowhurst R. N. Mockler T. C. Folkerts O. Delcher A. L.. (2011). The genome of woodland strawberry (Fragaria vesca). Nat. Genet. 43, 109–116. doi: 10.1038/ng.740 21186353 PMC3326587

[B33] Sievers F. Higgins D. G. Russell D. J. (2014). “Multiple sequence alignment methods,” in "Clustal omega, accurate alignment of very Large numbers of sequences," (Totowa, NJ: Humana Press), 105–116. 10.1007/978-1-62703-646-7_6 24170397

[B34] Song C. Liu Y. Song A. Dong G. Zhao H. Sun W.. (2018). The chrysanthemum nankingense genome provides insights into the evolution and diversification of chrysanthemum flowers and medicinal traits. Mol. Plant 11, 1482–1491. doi: 10.1016/j.molp.2018.10.003 30342096

[B35] Song X. Wang J. Li N. Yu J. Meng F. Wei C.. (2020). Deciphering the high-quality genome sequence of coriander that causes controversial feelings. Plant Biotechnol. J. 18, 1444–1456. doi: 10.1111/pbi.13310 31799788 PMC7206992

[B36] Steuernagel B. Witek K. Krattinger S. G. Ramirez-Gonzalez R. H. Schoonbeek H.-J. Yu G.. (2020). The NLR-annotator tool enables annotation of the intracellular immune receptor Repertoire1 [OPEN]. Plant Physiol. 183, 468–482. doi: 10.1104/pp.19.01273 32184345 PMC7271791

[B37] Wickham H. (2016). ggplot2: elegant graphics for data analysis (Springer).

[B38] Wu Z. Tian L. Liu X. Huang W. Zhang Y. Li X. (2022). The n-terminally truncated helper NLR NRG1C antagonizes immunity mediated by its full-length neighbors NRG1A and NRG1B. Plant Cell 34, 1621–1640. doi: 10.1093/plcell/koab285 34871452 PMC9048947

[B39] Xin X.-F. Kvitko B. He S. Y. (2018). Pseudomonas syringae: what it takes to be a pathogen. Nat. Rev. Microbiol. 16, 316–328. doi: 10.1038/nrmicro.2018.17 29479077 PMC5972017

[B40] Xu S. Dai Z. Guo P. Fu X. Liu S. Zhou L.. (2021). ggtreeExtra: Compact visualization of richly annotated phylogenetic data. Mol. Biol. Evol. 38, 4039–4042. doi: 10.1093/molbev/msab166 34097064 PMC8382893

[B41] Yang T. Liu R. Luo Y. Hu S. Wang D. Wang C.. (2022). Improved pea reference genome and pan-genome highlight genomic features and evolutionary characteristics. Nat. Genet. 54, 1553–1563. doi: 10.1038/s41588-022-01172-2 36138232 PMC9534762

[B42] Yang J. Liu D. Wang X. Ji C. Cheng F. Liu B.. (2016). The genome sequence of allopolyploid brassica juncea and analysis of differential homoeolog gene expression influencing selection. Nat. Genet. 48, 1225–1232. doi: 10.1038/ng.3657 27595476

[B43] Yang H. Sun Y. Wang H. Zhao T. Xu X. Jiang J.. (2021). Genome-wide identification and functional analysis of the ERF2 gene family in response to disease resistance against stemphylium lycopersici in tomato. BMC Plant Biol. 21, 72. doi: 10.1186/s12870-021-02848-3 33530947 PMC7856819

[B44] Yao L. Yang B. Ma X. Wang S. Guan Z. Wang B.. (2020). A genome-wide view of transcriptional responses during aphis glycines infestation in soybean. Int. J. Mol. Sci. 21, 5191. doi: 10.3390/ijms21155191 32707968 PMC7432633

[B45] Yu G. Smith D. K. Zhu H. Guan Y. Lam T. T.-Y. (2017). Ggtree: an r package for visualization and annotation of phylogenetic trees with their covariates and other associated data. Methods Ecol. Evol. 8, 28–36. doi: 10.1111/2041-210X.12628

[B46] Zhang W. Yuan Q. Wu Y. Zhang J. Nie J. (2022). Genome-wide identification and characterization of the CC-NBS-LRR gene family in cucumber (Cucumis sativus l.). Int. J. Mol. Sci. 23, 5048. doi: 10.3390/ijms23095048 35563438 PMC9099878

[B47] Zhao W. Liu Y. Li L. Meng H. Yang Y. Dong Z.. (2021). Genome-wide identification and characterization of bHLH transcription factors related to anthocyanin biosynthesis in red walnut (Juglans regia l.). Front. Genet. 12. doi: 10.3389/fgene.2021.632509 PMC7943622 33719341

