# Supplementary figures and images for: PlantNLRatlas: a comprehensive dataset of full- and partial-length NLR resistance genes across 100 chromosome-level plant genomes
Source: Front Plant Sci. 2023 Apr 14;14:1178069. doi: 10.3389/fpls.2023.1178069 (PMC10146310; doi:10.3389/fpls.2023.1178069)

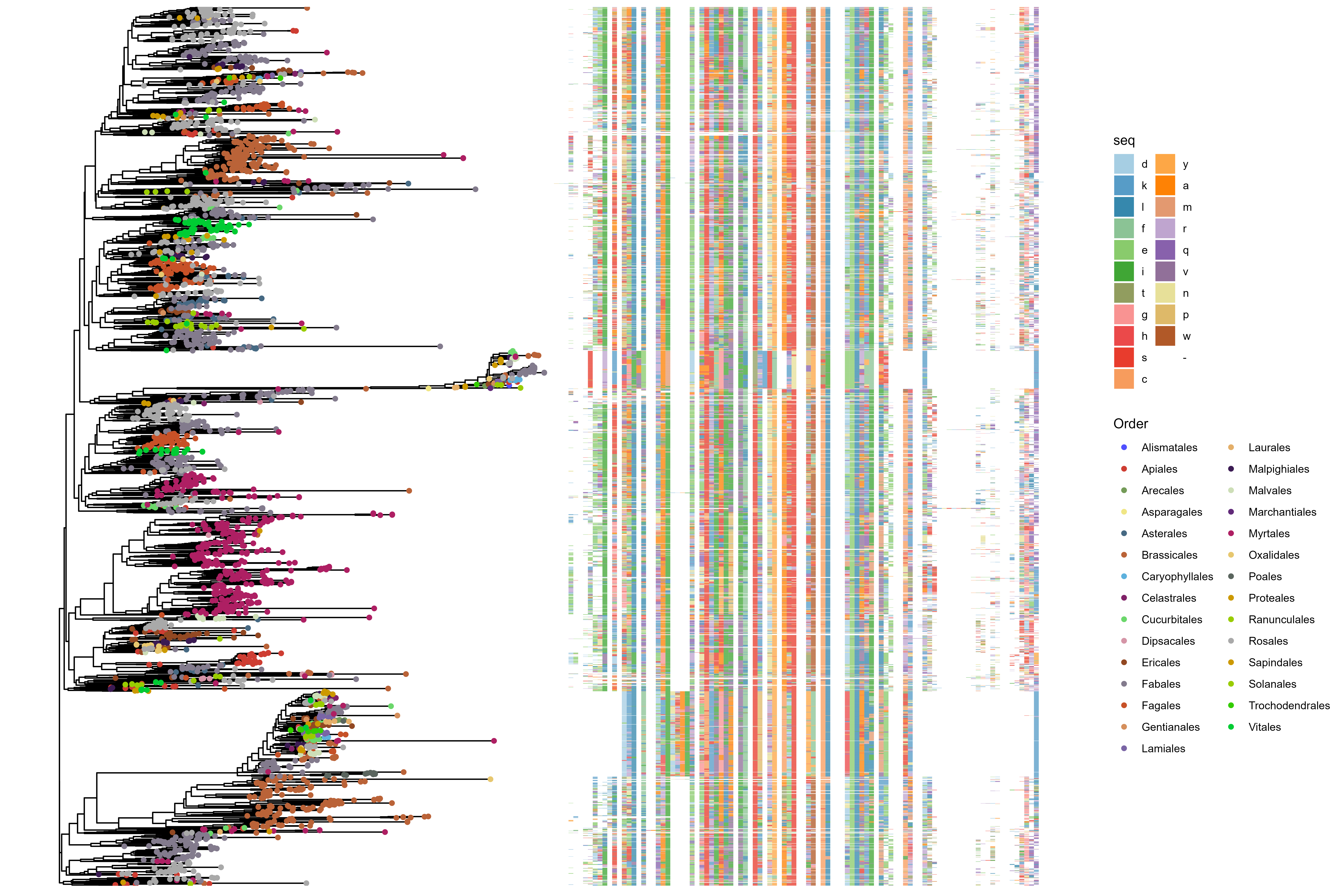

Supplement: Supplementary Figure 2 — Phylogenetic analysis of group T. The left panel shows the phylogenetic tree created using FastTree, with different colors denoting different orders. The right panel shows the corresponding aligned domain sequences, with different colors corresponding to different amino acids. [file Image_2.png]

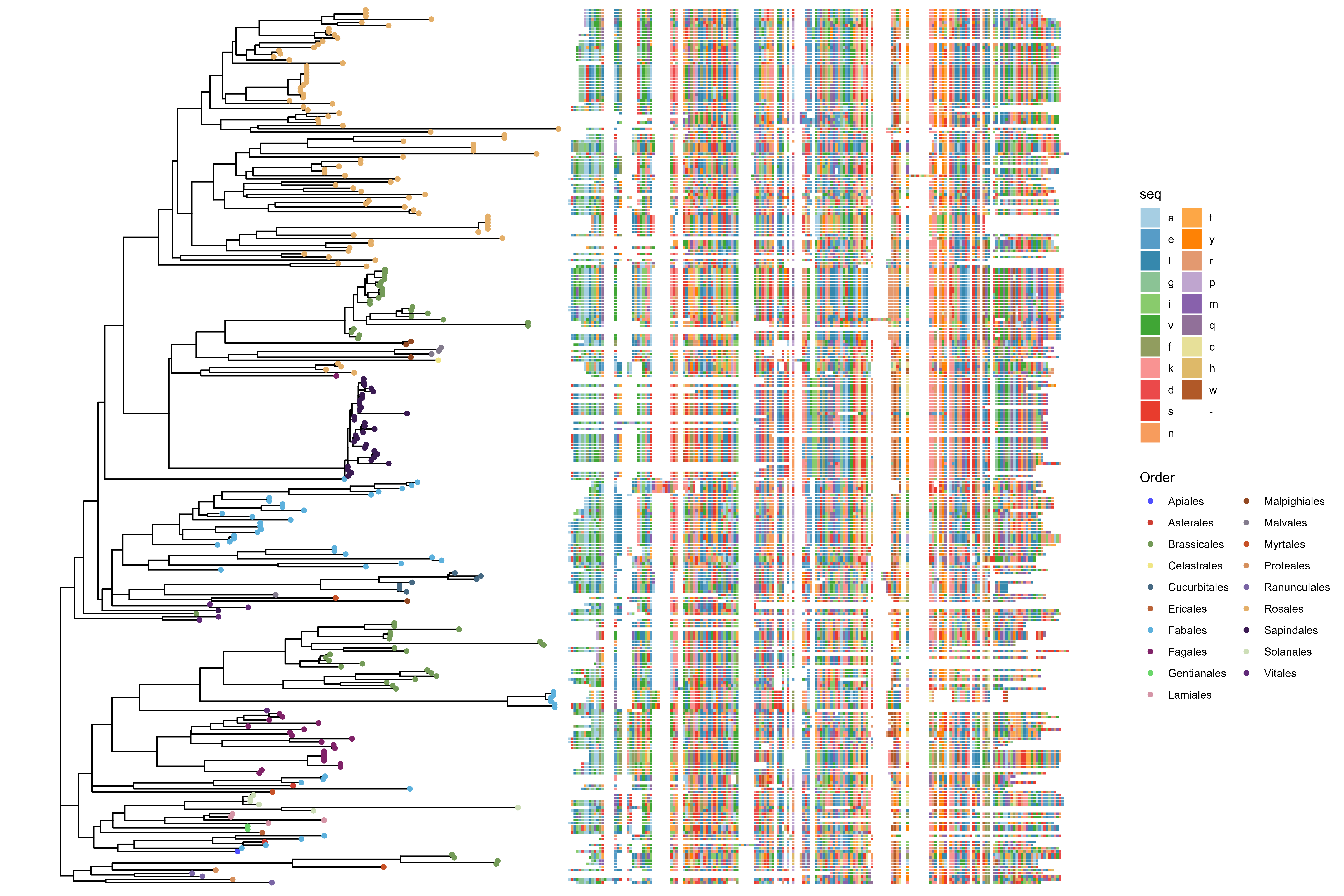

Supplement: Supplementary Figure 3 — Phylogenetic analysis of group R. The left panel shows the phylogenetic tree created using FastTree, with different colors denoting different orders. The right panel shows the corresponding aligned domain sequences, with different colors corresponding to different amino acids. [file Image_3.png]

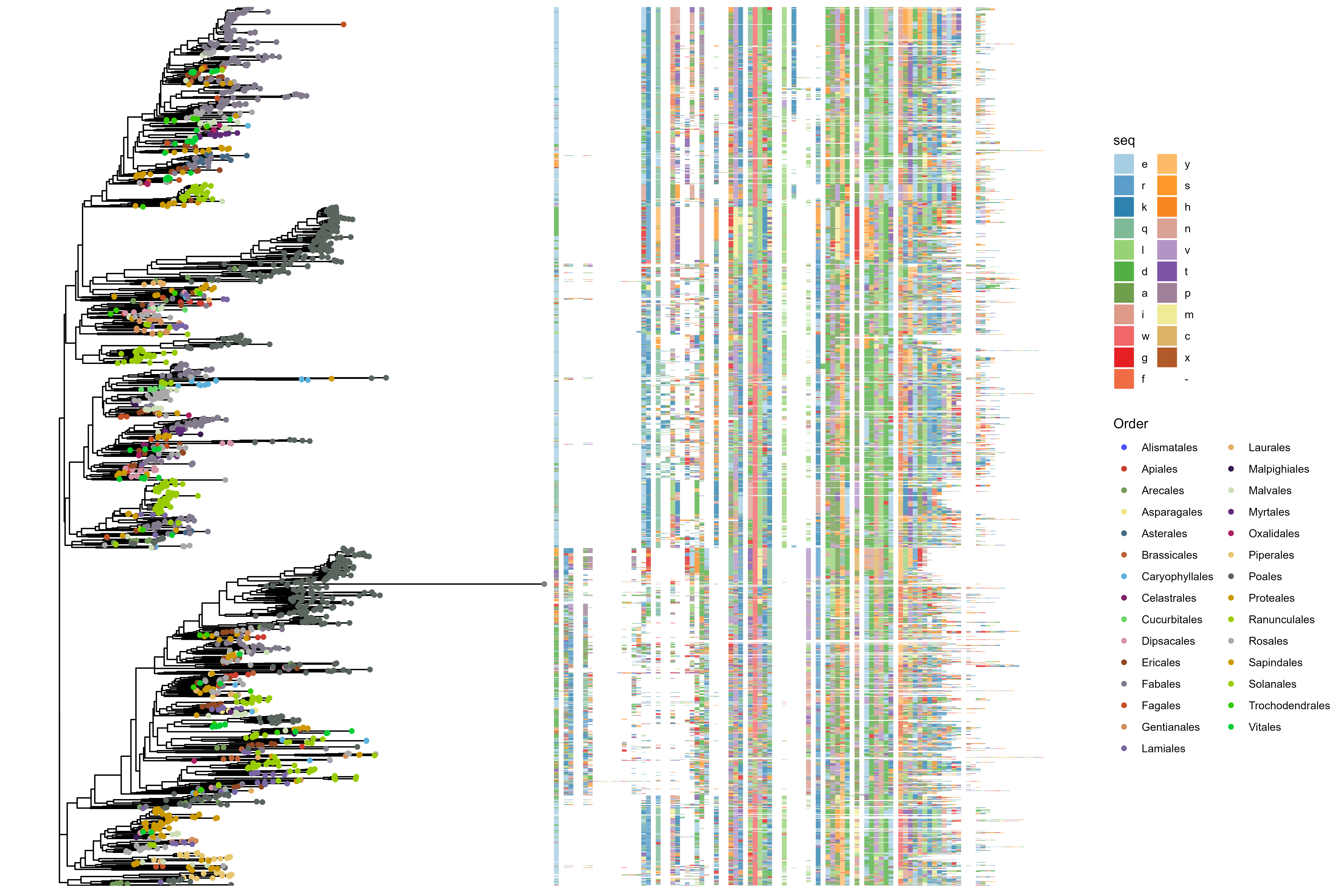

Supplement: Supplementary Figure 4 — Phylogenetic analysis of group C. The left panel shows the phylogenetic tree created using FastTree, with different colors denoting different orders. The right panel shows the corresponding aligned domain sequences, with different colors corresponding to different amino acids. [file Image_4.png]

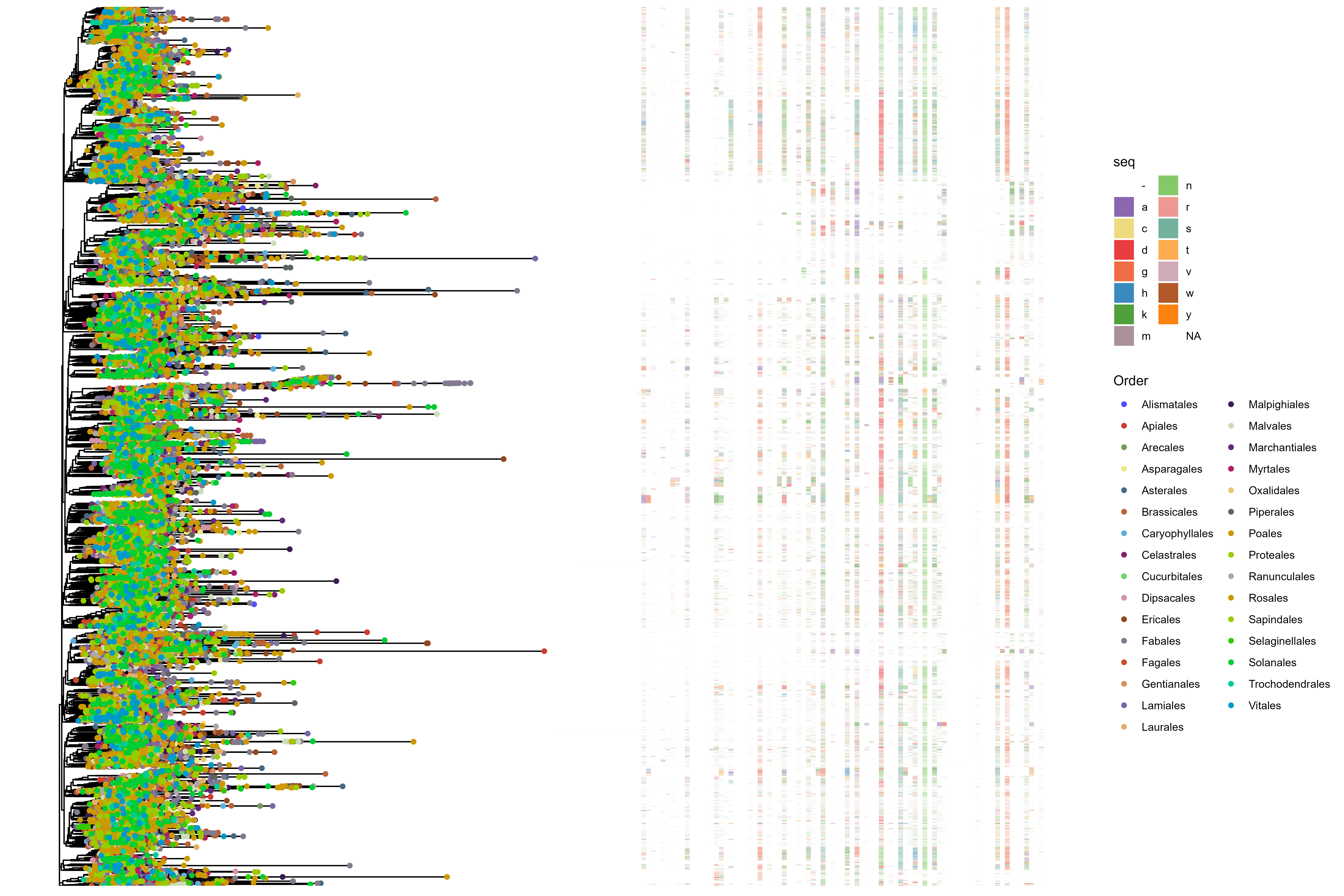

Supplement: Supplementary Figure 5 — Phylogenetic analysis of group L. The left panel shows the phylogenetic tree created using FastTree, with different colors denoting different orders. The right panel shows the corresponding aligned domain sequences, with different colors corresponding to different amino acids. [file Image_5.png]

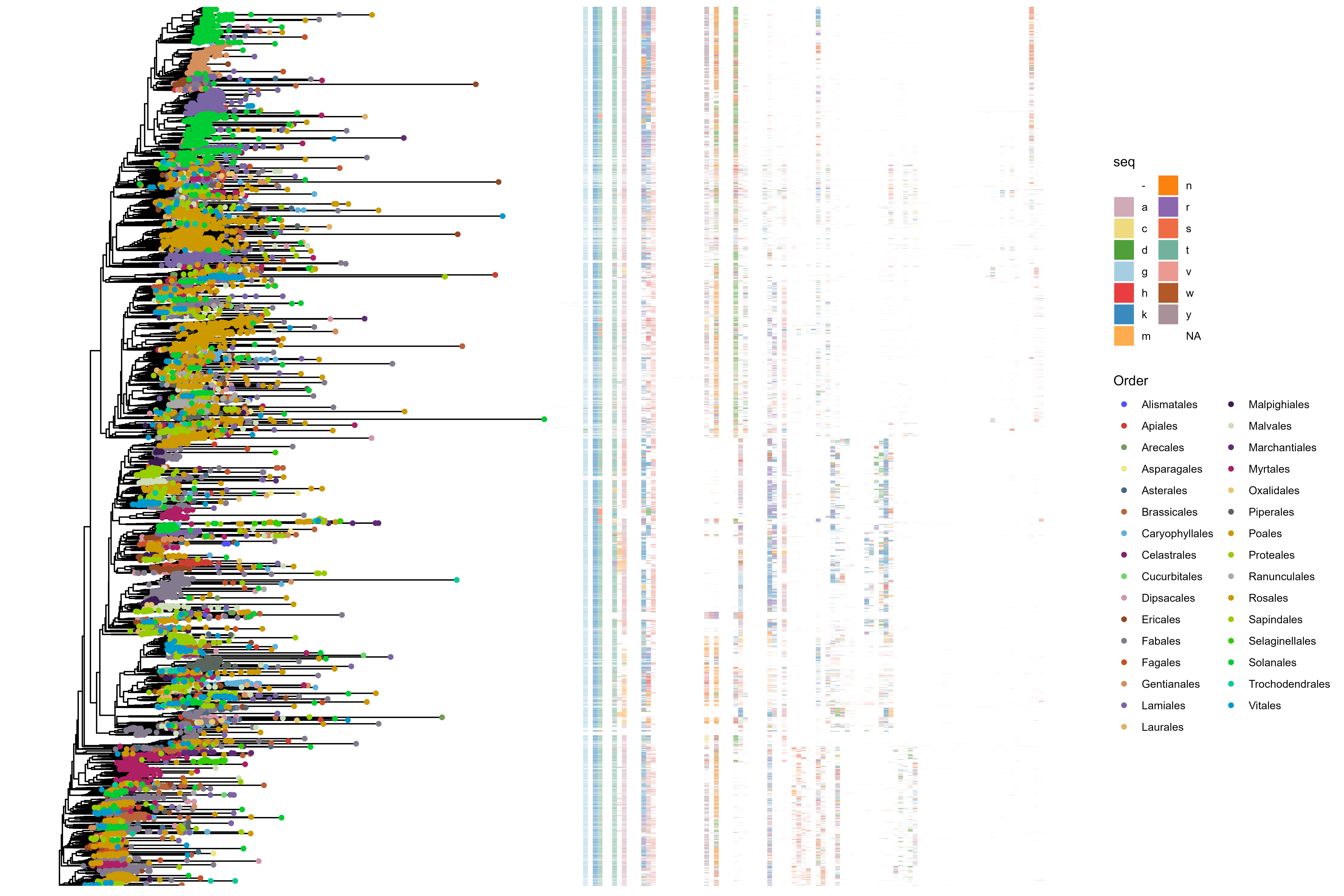

Supplement: Supplementary Figure 6 — Phylogenetic analysis of group N. The left panel shows the phylogenetic tree created using FastTree, with different colors denoting different orders. The right panel shows the corresponding aligned domain sequences, with different colors corresponding to different amino acids. [file Image_6.png]

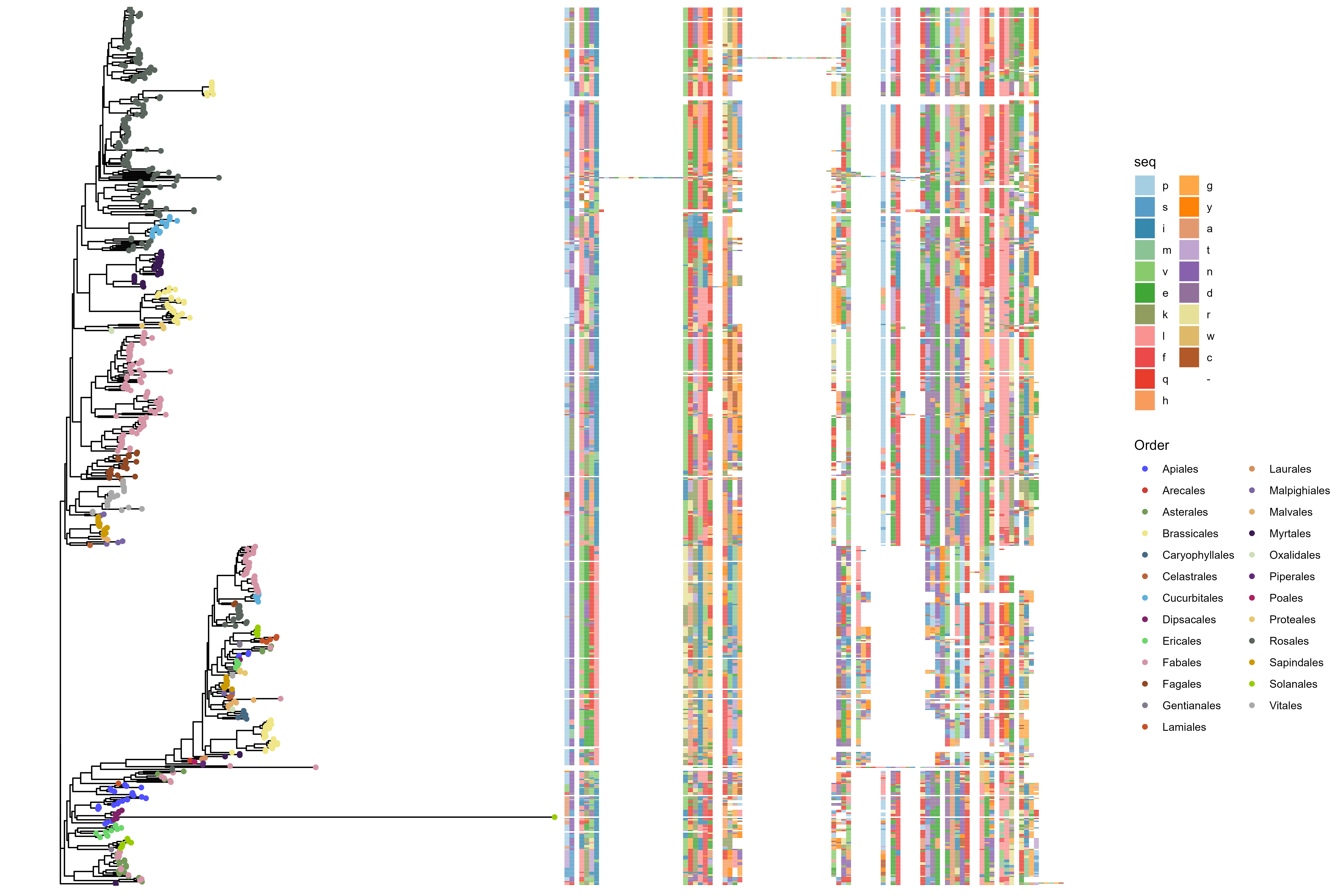

Supplement: Supplementary Figure 7 — Phylogenetic analysis of group RN. The left panel shows the phylogenetic tree created using FastTree, with different colors denoting different orders. The right panel shows the corresponding aligned domain sequences, with different colors corresponding to different amino acids. [file Image_7.png]

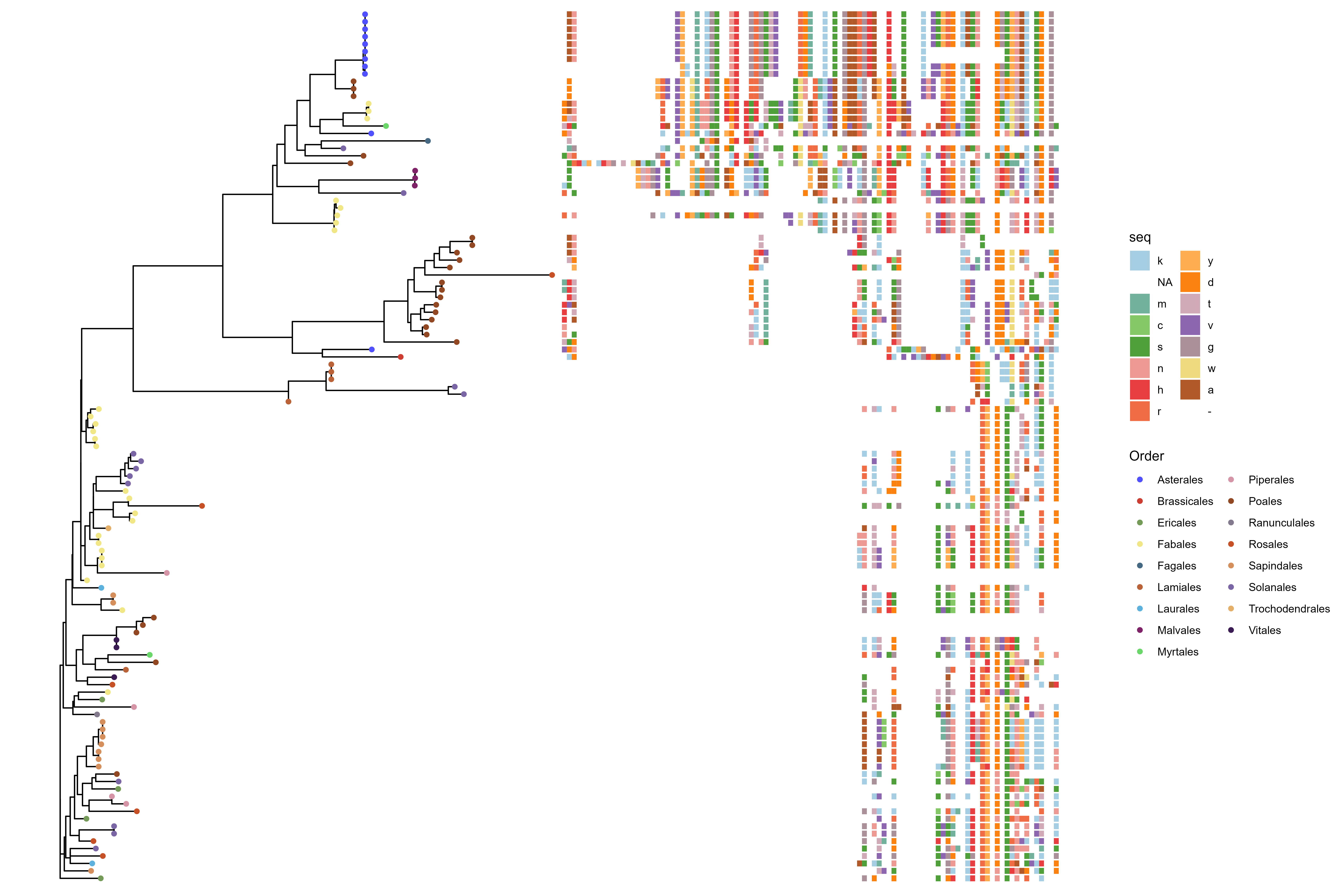

Supplement: Supplementary Figure 8 — Phylogenetic analysis of group CL. The left panel shows the phylogenetic tree created using FastTree, with different colors denoting different orders. The right panel shows the corresponding aligned domain sequences, with different colors corresponding to different amino acids. [file Image_8.png]

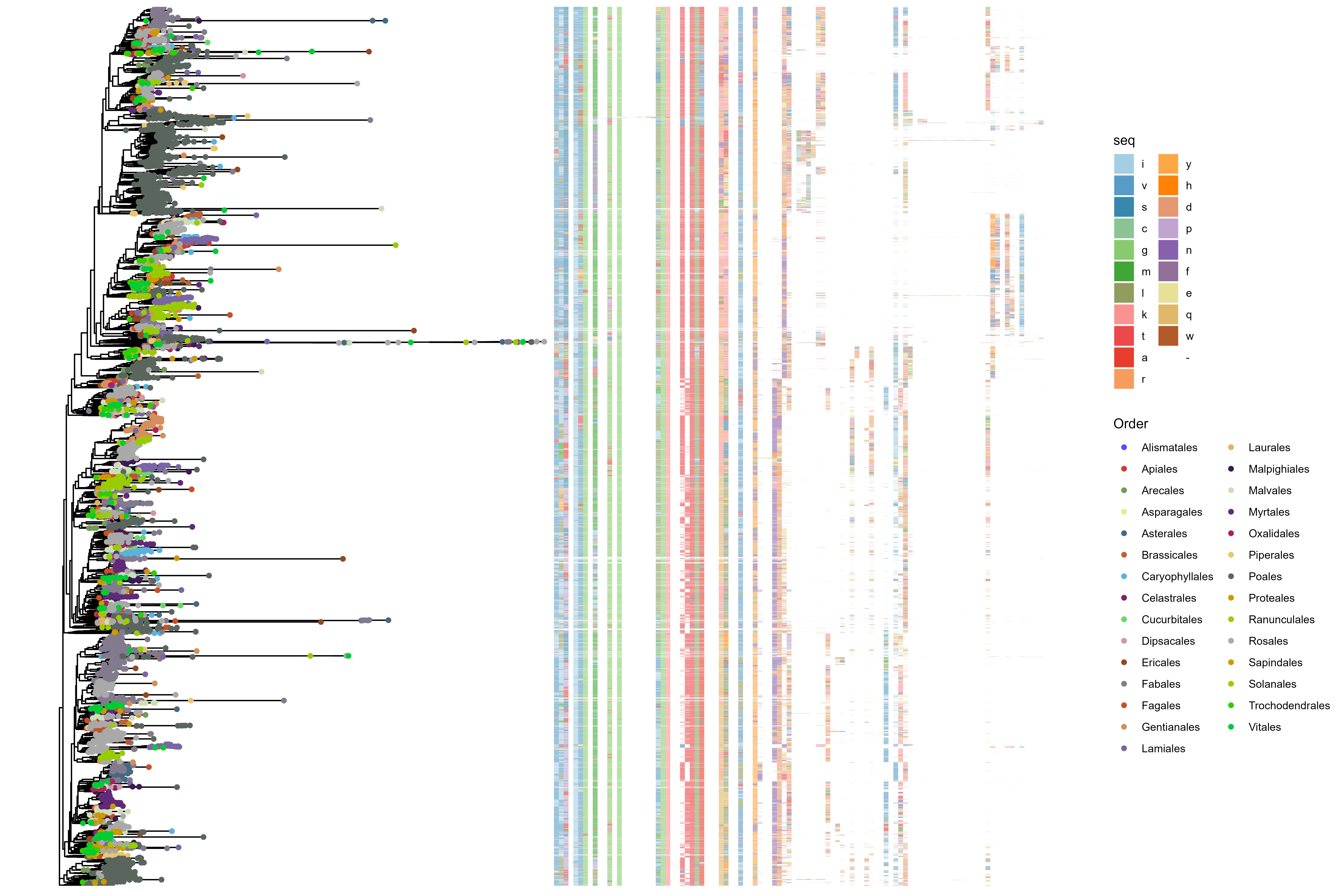

Supplement: Supplementary Figure 9 — Phylogenetic analysis of group CN. The left panel shows the phylogenetic tree created using FastTree, with different colors denoting different orders. The right panel shows the corresponding aligned domain sequences, with different colors corresponding to different amino acids. [file Image_9.png]

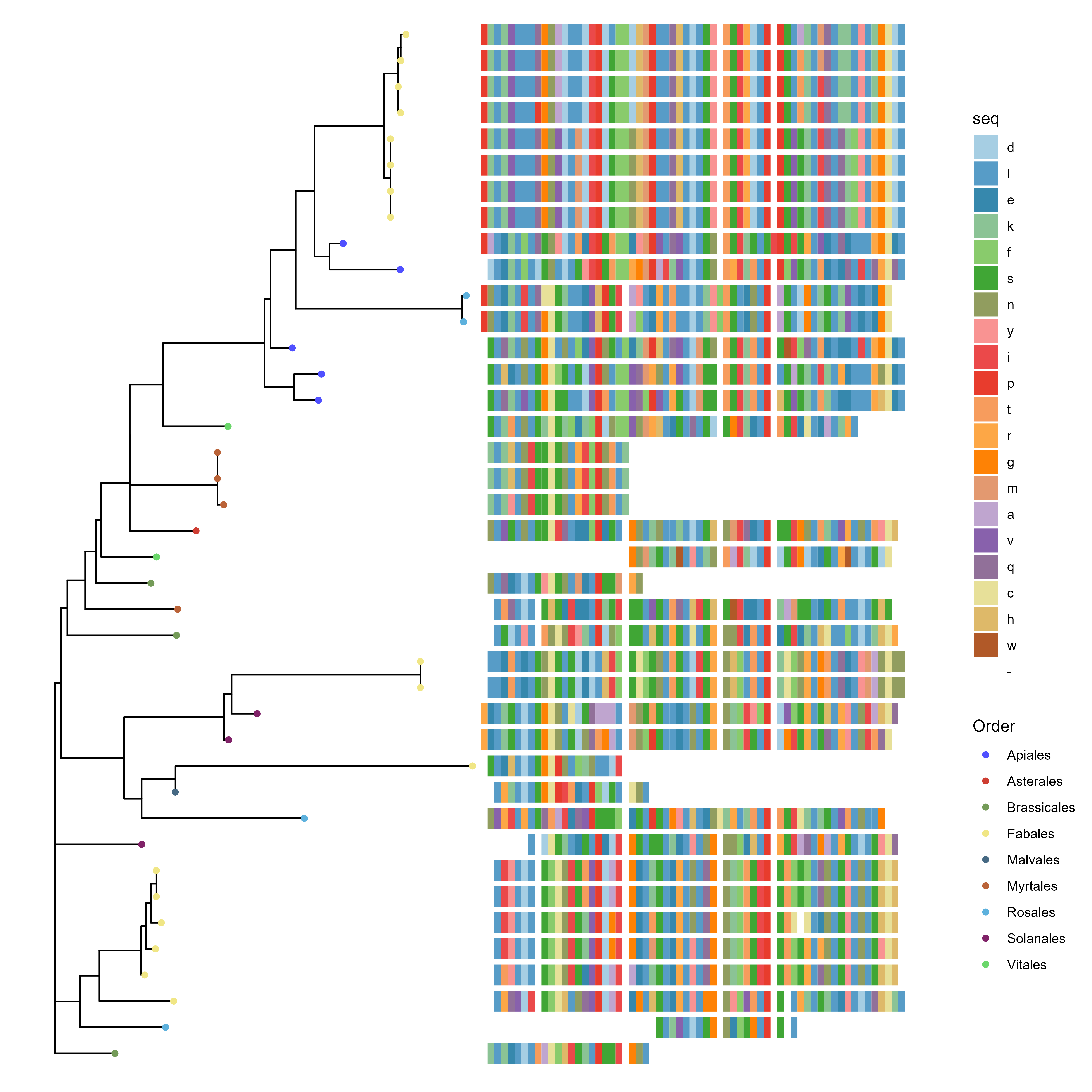

Supplement: Supplementary Figure 10 — Phylogenetic analysis of group TL. The left panel shows the phylogenetic tree created using FastTree, with different colors denoting different orders. The right panel shows the corresponding aligned domain sequences, with different colors corresponding to different amino acids. [file Image_10.png]

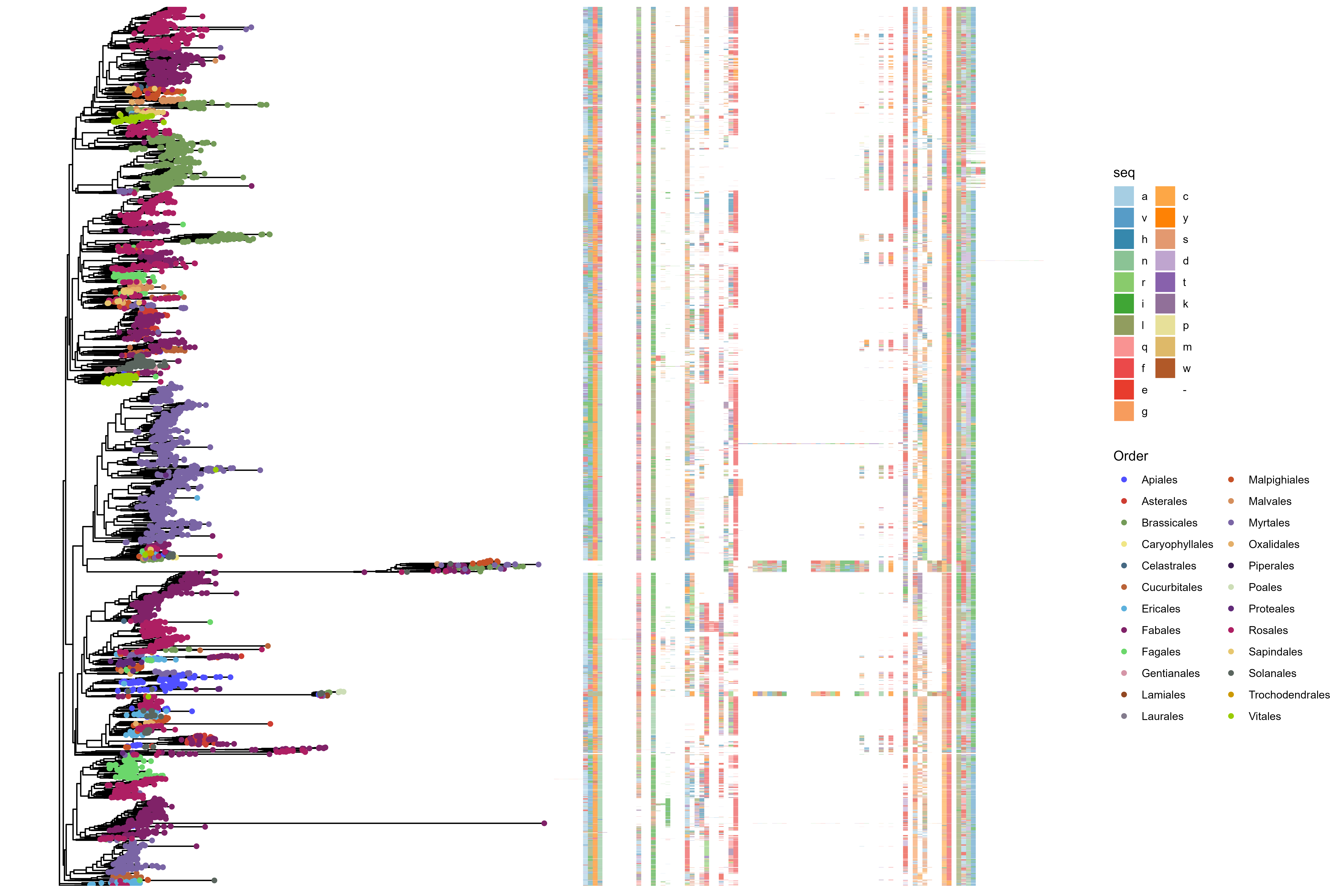

Supplement: Supplementary Figure 11 — Phylogenetic analysis of group TN. The left panel shows the phylogenetic tree created using FastTree, with different colors denoting different orders. The right panel shows the corresponding aligned domain sequences, with different colors corresponding to different amino acids. [file Image_11.png]

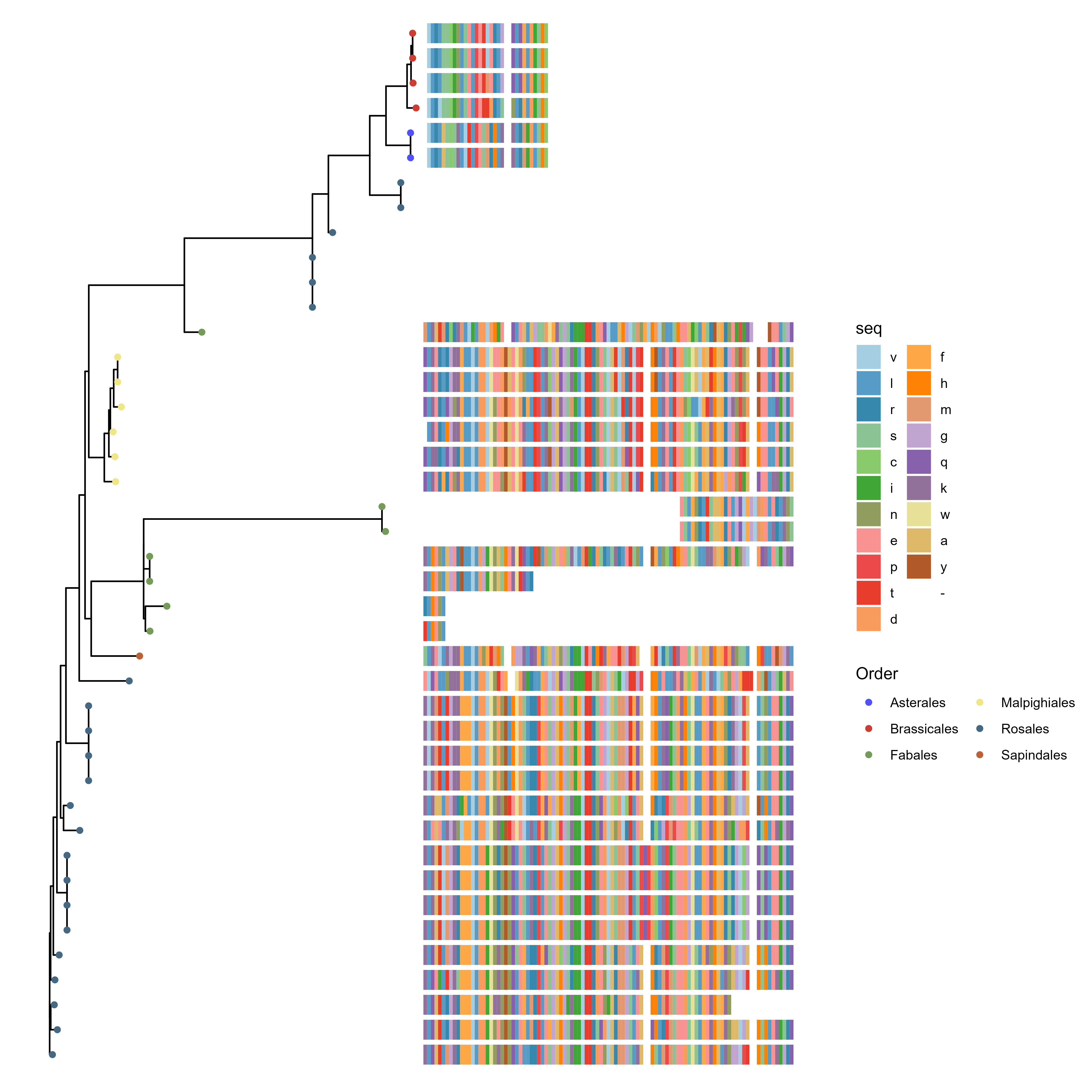

Supplement: Supplementary Figure 12 — Phylogenetic analysis of group RNL. The left panel shows the phylogenetic tree created using FastTree, with different colors denoting different orders. The right panel shows the corresponding aligned domain sequences, with different colors corresponding to different amino acids. [file Image_12.png]
